# A Quasi-experimental Study of Optimized Retractor Management on the Incidence of Femoral Nerve Injury Following Gynecological Surgery

**DOI:** 10.7759/cureus.75610

**Published:** 2024-12-12

**Authors:** Yukiko Omura, Kohei Ikeda, Mai Nabatame, Ichiro Kondo

**Affiliations:** 1 Anesthesiology, Jikei University School of Medicine, Tokyo, JPN

**Keywords:** femoral nerve injury, gynecological surgery, iatrogenic nerve complications, retractor-associated femoral nerve injury, retractor management

## Abstract

Background

Femoral neuropathy is a significant postoperative complication in gynecological surgery that can severely impact patient mobility and quality of life. Among various mechanisms of nerve injury, retractor-induced compression against the pelvic sidewall has been identified as a particularly crucial causative factor. Despite this well-recognized mechanism and its clinical importance, few studies have investigated specific preventive strategies for this iatrogenic complication.

Objective

This study aimed to evaluate the effectiveness of standardized modifications in retractor management protocols for reducing the incidence of retractor-associated femoral nerve injury following gynecological surgery for benign tumors.

Methods

This single-center quasi-experimental study compared two consecutive cohorts of patients undergoing gynecological surgery for benign tumors between February 2018 and July 2019. Women aged ≥20 years who received combined general and epidural anesthesia were eligible. The study consisted of two phases: an eight-month pre-intervention phase evaluating femoral nerve injury incidence under conventional retractor management (n = 107), followed by a 10-month post-intervention phase (n = 108) assessing the impact of three standardized protocol modifications, i.e., optimization of a hand-held retractor insertion depth, mandatory 30-minute intervals for retractor release, and conversion to non-metallic self-retaining abdominal wall retraction systems.

Results

Implementation of modified retractor management protocols resulted in a significant reduction in the incidence of femoral nerve injury from 6.5% (7/107 patients) to 0.9% (1/108 patients) (P = 0.035), with an odds ratio of 0.134 (95% CI: 0.012-0.807). All cases presented with unilateral neurological deficits confined to femoral and lateral femoral cutaneous nerve distributions, manifesting as quadriceps and iliopsoas muscle weakness with associated sensory disturbances. Complete neurological recovery was achieved in all cases within one to 12 months (mean duration: 5.5 ± 3.9 months).

Conclusions

This study demonstrates that implementing standardized modifications in retractor management protocols significantly reduces the risk of femoral nerve injury following gynecological surgery for benign tumors. The combination of optimized retractor depth, standardized release intervals, and non-metallic retraction systems provides a practical and effective approach to preventing this significant surgical complication. Future studies incorporating quantitative measures of retractor positioning and pressure may help establish standardized guidelines for optimal retractor management.

## Introduction

Postoperative neuropathy, particularly femoral neuropathy, is a significant complication following gynecological surgery that can lead to substantial morbidity and a devastating impact on patients' mobility and quality of life [1,2]. Among postoperative complications of gynecological surgery, retractor-associated femoral nerve injury represents a particularly concerning adverse event due to its relatively high incidence and potential for prolonged functional disability, significantly impacting patient mobility and quality of life [3]. The reported incidence of femoral nerve injury following gynecological surgery has varied in previous studies, with rates ranging from 1.1-1.9% [4,5] to approximately 8% when self-retaining metallic retractors were used [6]. Some earlier reports suggested rates as high as 11% [7], highlighting the need for standardized preventive interventions.

Previous studies have demonstrated that sustained compression and inadequate positioning of retractor blades represent major etiological factors in iatrogenic nerve injuries during gynecological surgery [8,9]. Furthermore, mechanical stress induced by suboptimal retractor placement, especially during prolonged surgical duration, has been established as a primary mechanism of direct nerve compression, resulting in postoperative neurological sequelae in benign gynecologic surgery [7,10].

Through our postoperative pain management service, we often identified cases of retractor-associated femoral neuropathy in patients following gynecological surgery for benign tumors, which were distinct from the transient neurological symptoms associated with epidural anesthesia. To reduce the incidence of retractor-associated femoral nerve injury during gynecological surgery for benign tumors, we implemented modifications to intraoperative retractor management protocols: minimizing a hand-held retractor insertion depth, limiting the duration of continuous tissue compression, and utilizing non-metallic self-retaining retractor systems. We hypothesized that standardized modifications in intraoperative retractor management protocols would significantly reduce the incidence of femoral nerve injury following gynecologic surgery for benign tumors. To address this hypothesis, we conducted a quasi-experimental study comparing two consecutive cohorts of patients who underwent gynecologic surgery. The study consisted of two phases: an eight-month pre-intervention phase evaluating the incidence of retractor-associated femoral injury under conventional retractor management, followed by a 10-month post-intervention phase assessing the impact of three specific protocol modifications, i.e., optimization of a hand-held retractor depth, standardization of intermittent retractor release, and implementation of non-metallic self-retaining abdominal wall retraction systems.

## Materials and methods

The study protocol was approved by the Jikei University Institutional Review Board (30-333{9354}). This quasi-experimental study employed a before-and-after design to evaluate the effectiveness of preventive interventions. We conducted a retrospective review of medical records to examine the incidence of femoral nerve injury in patients who underwent surgery for gynecological benign tumors at Jikei University Hospital, Tokyo, Japan. The study included women aged ≥20 years who received combined general and epidural anesthesia for their procedures between February and September 2018. Exclusion criteria included laparoscopic procedures, malignant pathology, preexisting lower extremity neuropathy, and absence of epidural anesthesia. Following this initial analysis, three standardized modifications in retractor management protocols were implemented, and the incidence of postoperative femoral nerve injury was evaluated between October 2018 and July 2019.

All surgical procedures were performed with patients in either a supine or lithotomy position. An epidural catheter was placed at the level of Th (thoracic) 10/11 or Th11/12 and continuous epidural infusion of 0.08% levobupivacaine was initiated at a rate of 2-6 mL/hour. The postoperative pain management team conducted systematic neurological examinations of all patients to detect lower extremity neuropathy. For patients presenting with sensorimotor deficits in the lower extremities, epidural infusion was initially discontinued to evaluate the potential resolution of symptoms. When neurological symptoms persisted, we conducted a comprehensive neurological assessment, including spine magnetic resonance imaging, to differentiate between epidural anesthesia-related symptoms and intraoperative femoral nerve injury.

We compared the incidence of retractor-associated femoral nerve injury before and after the implementation of the retractor management protocol modifications and evaluated their effectiveness in reducing iatrogenic femoral nerve injury. In addition, we analyzed the temporal course of neurological recovery through a retrospective chart review. The implemented protocol modifications in this study consisted of three standardized interventions: optimization of a hand-held retractor insertion depth by reducing blade length from 12 cm to 9 cm, establishment of mandatory 30-minute intervals for retractor release with a minimum duration of several minutes per release, and conversion from conventional metallic retractors to non-metallic self-retaining abdominal wall retraction systems made of medical-grade plastic resin.

The selection of 30-minute intervals for mandatory hand-held retractor release was based on previous research by Warner et al., who demonstrated that lithotomy positions maintained for less than 30 minutes were not associated with peripheral nerve injuries [11]. The 30-minute intervals were managed by operating room nurses who reminded surgeons to assess for possible retractor release. While the immediate release was not always feasible due to critical surgical steps, surgeons were instructed to loosen the retractors when safely possible. The duration of release was at the surgeon's discretion, with a requirement of a minimum of several minutes for each release.

Sample size calculation was performed based on previously reported incidence rates [3,6,7,9]. Assuming a baseline incidence of 8% and expecting a reduction to 1% with preventive intervention, we calculated that 110 patients per group (including 10% to account for potential dropouts) would provide 80% power to detect this difference with a two-sided alpha of 0.05. Data analysis was performed using Prism (GraphPad Software, La Jolla, CA). Continuous variables are presented as mean ± standard deviation (SD). Between-group comparisons were performed using Student's t-test for continuous variables and Fisher's exact test for categorical variables. A two-sided p-value <0.05 was considered statistically significant.

## Results

A total of 215 patients were enrolled in this study; 107 patients underwent gynecological surgery for benign tumors before implementation of the modified retractor management protocols and 108 patients underwent surgery after implementation. Baseline demographic and surgical characteristics are summarized in Table 1. No significant differences were observed between the pre- and post-implementation groups with respect to age, height, weight, body mass index (BMI), American Society of Anesthesiologists Physical Status (ASA-PS), duration of surgery, duration of anesthesia, and intraoperative blood loss.

**Table 1:** Comparison of patient characteristics and operative variables between the pre- and post-implementation groups. BMI: body mass index, ASA-PS: American Society of Anesthesiologists Physical Status

	Pre- implementation (n = 107)	Post-implementation (n = 108)	P value
Age (years)	45±11	44±11	0.54
Height (cm)	158±6	159±5	0.42
Body weight (kg)	57±11	57±10	0.75
BMI (kg/m^2^)	23±4	22±4	0.32
ASA-PS 1/2/3	61/44/2	58/49/1	0.71
Duration of surgery (min)	143±53	143±50	0.99
Duration of anesthesia (min)	198±55	197±54	0.91
Intra-operative blood loss (ml)	365±613	302±373	0.36

**Figure 1:**
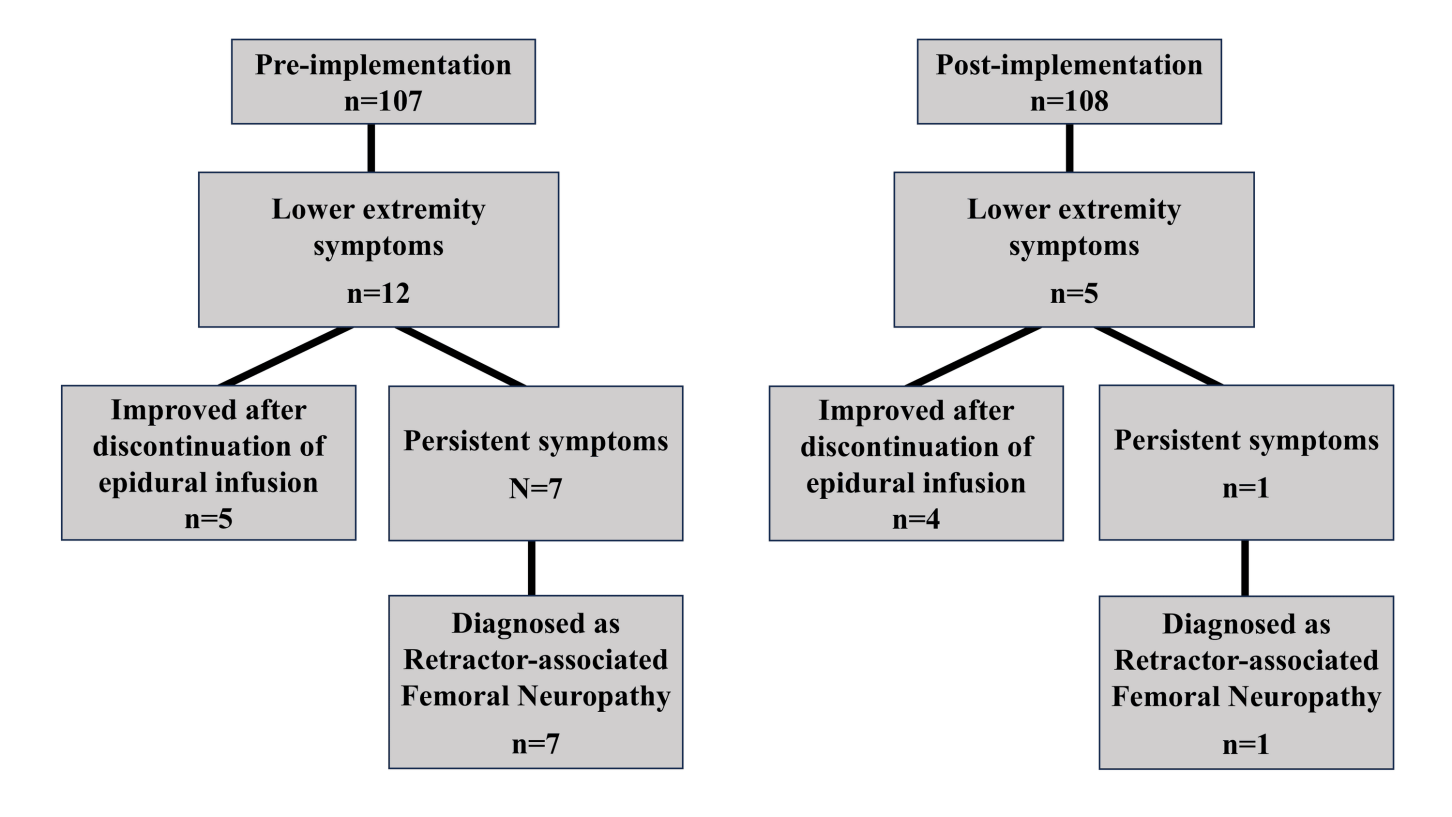
Diagnostic workflow for postoperative lower extremity neurological symptoms

Figure 1 shows the diagnostic workflow for postoperative lower-extremity neurological symptoms. Among 107 pre-implementation patients, 12 developed symptoms, with five showing improvement after discontinuation of epidural infusion and seven diagnosed with retractor-associated femoral neuropathy. In the post-implementation group (n = 108), five patients developed symptoms, with four improving after discontinuation of epidural infusion and one diagnosed with retractor-associated femoral neuropathy. Implementation of the modified retractor management protocols resulted in a significant reduction in the incidence of retractor-associated femoral nerve injury (Table 2). The incidence decreased from 6.5% (7/107 patients) in the pre-implementation group to 0.9% (1/108 patients) in the post-implementation group (P = 0.035), with an odds ratio (OR) of 0.134 (95% CI: 0.012-0.807) for developing femoral nerve injury after protocol implementation. Intraoperative hemodynamic parameters were maintained within normal ranges in all cases, with no episodes of critical hypotension that could potentially contribute to ischemic nerve injury.

**Table 2:** Incidence of retractor-associated femoral nerve injury before and after the implementation of the modified retractor protocols.

	Pre-implementation	Post-implementation	P-value	Odds ratio (95% CI)
Postoperative nerve injury: n (%)	7 (6.5%)	1 (0.9%)	0.035	0.134 (0.012-0.807)

In all cases diagnosed with retractor-associated nerve injury, neurological deficits were confined to femoral and lateral femoral cutaneous nerve distributions. Clinical examination revealed characteristic patterns: motor deficits manifesting as quadriceps and iliopsoas muscle weakness, accompanied by sensory disturbances in the anterior and lateral aspects of the thigh. Eight cases of retractor-associated femoral neuropathy were identified in this study (Table 3). Of these, seven occurred in the pre-implementation phase and one in the post-implementation phase. Clinical presentations were consistently unilateral, with the majority of cases (7/8) occurring after hysterectomy. The predominant manifestations included motor deficits affecting the quadriceps and iliopsoas muscles, accompanied by sensory disturbances along the anteromedial or lateral aspects of the thigh. These patterns and distribution of neurological deficits were consistent with femoral and lateral femoral cutaneous nerve involvement. All affected patients achieved complete neurological recovery, with recovery times ranging from one to 12 months (mean duration: 5.5 ± 3.9 months).

**Table 3:** Clinical presentations and outcomes of retractor-associated femoral nerve injury in eight cases. BMI: body mass index, Epi; epidural catheter insertion level, Th; thoracic, Ope duration: duration of operation

	Procedure	Patient characteristics and operative variables	Clinical presentation	Outcome
Pre-implementation				
Case 1	Hysterectomy, salpingo-oophorectomy	Age: 47, BMI: 24.8, Epi: Th 11/12, Ope duration: 97 min, blood loss: 190 ml, position: lithotomy, incision: transverse, weight of specimen: uterus, fallopian tube, 443 g	Unilateral weakness of the iliopsoas and quadriceps muscles, hypoesthesia along the inguinal region to the anterior thigh	Near complete recovery in 12 months
Case 2	Hysterectomy	Age: 49, BMI: 19.4, Epi: Th 10/11, Ope duration: 173 min, blood loss: 755 ml, position: supine, incision: vertical, weight of specimen: uterus, fallopian tube, 878g	Unilateral weakness of the iliopsoas and quadriceps muscles, hypoesthesia along the inguinal region to the anterior thigh	Near complete recovery in three months
Case 3	Hysterectomy, salpingo-oophorectomy	Age: 43, BMI: 30.5, Epi: Th 11/12, Ope duration: 120 min, blood loss: 375 ml, position: lithotomy, incision: vertical, weight of specimen: uterus, fallopian tube, 176 g	Unilateral weakness of the quadriceps muscle, hypoesthesia along the anterior thigh	Near complete recovery in three months
Case 4	Hysterectomy, salpingo-oophorectomy	Age: 41, BMI: 20.7, Epi: Th 11/12, Ope duration: 132 min, blood loss: 350 ml, position: supine, incision: vertical, weight of specimen: uterus, fallopian tube, 594 g	Unilateral weakness of the quadriceps muscle, hypoesthesia along the anteromedial thigh	Near complete recovery in three months
Case 5	Salpingo-oophorectomy	Age: 32, BMI: 21.4, Epi: Th 11/12, Ope duration: 112 min, blood loss: 130 ml, position: lithotomy, incision: vertical, weight of specimen: ovary, fallopian tube, 17 g	Unilateral hypoesthesia along the anteromedial thigh	Complete recovery in four months
Case 6	Hysterectomy, salpingo-oophorectomy	Age: 45, BMI: 24.1, Epi: Th 10/11, Ope duration: 96 min, blood loss: 630 ml, position: supine, incision: vertical, weight of specimen: uterus, ovary, fallopian tube, 789 g	Unilateral hypoesthesia along the anteromedial thigh	Complete recovery in one month
Case 7	Hysterectomy, salpingo-oophorectomy	Age: 51, BMI: 20.0, Epi: Th 11/12, Ope duration: 157 min, blood loss: 300 ml, position: supine, incision: transverse, weight of specimen: uterus, fallopian tube, 759 g	Unilateral weakness of the iliopsoas and quadriceps muscles, hypoesthesia along the anteromedial thigh and knee	Complete recovery in nine months
Post-implementation				
Case 8	Hysterectomy	Age: 45, BMI: 20.0, Epi: Th 11/12, Ope duration: 160 min, blood loss: 500 ml, position: supine, incision: transverse, weight of specimen: uterus, ovary, fallopian tube, 763 g	Unilateral weakness of the iliopsoas and quadriceps muscles, hypoesthesia along the lateral thigh	Complete recovery in nine months

## Discussion

Various types of nerve injuries have been reported following gynecological surgery, including damage to the ilioinguinal, iliohypogastric, obturator, femoral, genitofemoral, lateral femoral cutaneous, pudendal, sciatic, and common fibular nerves [11-14]. Among these potential complications, femoral neuropathy represents the most frequently reported iatrogenic nerve injury, with historical studies suggesting incidence rates as high as 11% [7]. The vulnerability of the femoral nerve during gynecological procedures is particularly notable due to its anatomical course along the pelvic sidewall, where it is susceptible to compression injury from improperly placed or managed retractors. Recent systematic reviews have highlighted the significant impact of femoral neuropathy on postoperative recovery and patient quality of life, emphasizing the importance of preventive strategies [15].

This quasi-experimental study demonstrated that standardized modifications in retractor management protocols significantly reduced the incidence of femoral nerve injury following gynecological surgery for benign disease. Implementation of three specific interventions - optimization of hand-held retractor depth, mandatory intermittent release, and conversion to non-metallic self-retaining retraction systems - resulted in a substantial reduction in the incidence of femoral nerve injury from 6.5% to 0.9%. This reduction is particularly noteworthy given our pre-intervention incidence rate of 6.5%, which was higher than the 1.1-1.9% reported in previous studies [4,5,16]. The significant decrease in risk (OR: 0.134; 95% CI: 0.012-0.807) suggests that standardized retractor management protocols can effectively prevent this significant surgical complication.

In this study, we implemented three specific modifications to standard retractor management protocols. First, we reduced the hand-held retractor blade length from 12 cm to 9 cm, which optimized insertion depth while still maintaining adequate surgical exposure. This modification was designed to minimize the risk of excessive pressure on the pelvic sidewall where the femoral nerve is vulnerable to compression injury. Second, we established mandatory 30-minute intervals for retractor release, based on Warner et al.'s findings that peripheral nerve injuries were not observed in procedures where tissue compression lasted less than 30 minutes [11]. During these release periods, which lasted several minutes at the surgeon's discretion, the retractor was completely disengaged from the tissue to allow for the recovery of compressed structures. Third, we replaced conventional metallic self-retaining abdominal wall retractors with non-metallic systems made of medical-grade plastic resin. This change was implemented based on Maneschi et al.'s observation of increased nerve injury rates associated with metallic retractors [3], particularly in cases using rigid metallic retraction systems. The plastic resin self-retaining retractors potentially offer more compliant tissue interaction and better pressure distribution.

The anatomical course of the femoral nerve explains its vulnerability to iatrogenic injury during gynecological surgery. Originating from the L2 to L4 nerve roots within the lumbar plexus, the nerve descends through the groove between the iliac and psoas major muscles before passing beneath the inguinal ligament to enter the thigh [17]. This anatomical pathway, particularly where the nerve courses along the pelvic sidewall, creates areas of potential compression during surgery [7]. The observed patterns of neurological deficits in our study - motor deficits affecting the quadriceps and iliopsoas muscles, combined with sensory disturbances in the anterior and lateral thigh - precisely correspond to this anatomical distribution.

Intraoperative nerve injury can occur through various mechanisms, including compression, stretch, entrapment, or transection of nerve fibers [16]. However, compression-induced ischemic damage has been identified as the predominant mechanism in retractor-associated femoral nerve injury [18]. This is particularly evident in cases involving self-retaining metallic retractors, where improper placement and excessive depth can create sustained pressure points against the pelvic sidewall [10,19]. The significance of retractor-induced injury is supported by a prospective study demonstrating an 8% incidence of femoral nerve injury with self-retaining metallic retractors, compared to less than 1% when alternative retraction methods were employed [6,9]. Our study focused exclusively on gynecological surgeries for benign conditions, which present distinct operative considerations compared to procedures for malignant disease. While malignant cases typically require wider incisions, benign procedures are often performed through smaller incisions, potentially leading to increased retractor force or tissue compression to achieve adequate surgical exposure. This mechanical difference may have contributed to the observed incidence and characteristics of retractor-associated femoral neuropathy in our study population. The effectiveness of our interventions - particularly the optimization of hand-held retractor depth and implementation of mandatory release intervals - supports the hypothesis that mechanical compression is indeed the primary mechanism of nerve injury in these cases. The conversion from metallic to non-metallic self-retaining abdominal wall retraction systems may reduce the risk of nerve injury through more optimal pressure distribution.

The natural history and recovery patterns of retractor-associated femoral neuropathy demonstrate a generally favorable prognosis. Previous literature has documented a predictable recovery timeline, with 25% of cases resolving within one week, another 25% within one month, 35% within five months, and the remaining 15% within one year [18]. Our findings align with these reported recovery patterns, as all patients in our study achieved complete neurological recovery within 12 months, with a mean recovery time of 5.5 months. This temporal course of recovery is consistent with the previous literature on postoperative peripheral nerve injuries following gynecologic surgery [16,20]. Notably, the recovery patterns were similar between pre- and post-implementation cases in our study, suggesting that while our modified protocols effectively prevented nerve injury, they did not alter the natural course of recovery once injury occurred. This observation has important clinical implications, emphasizing that prevention through proper retractor management should remain the primary focus, as even temporary neurological deficits can significantly impact patient recovery and postoperative quality of life. The consistency of recovery patterns across studies also provides valuable prognostic information for patient counseling and postoperative management planning.

Several limitations of this study warrant consideration. First, while our protocol modifications were systematically implemented, we lacked objective measures to verify retractor positioning and pressure release compliance during each procedure. Second, as all three interventions were implemented simultaneously, we cannot determine the relative contribution or effectiveness of each modification. In addition, the single-center, quasi-experimental design may limit the generalizability of our findings. Future studies incorporating quantitative measures of retractor positioning and pressure, along with stepped implementation of individual interventions, would address these limitations.

## Conclusions

This study demonstrated that standardized modifications in retractor management protocols significantly reduced the incidence of femoral nerve injury following gynecological surgery for benign tumors. The implementation of optimized hand-held retractor depth, mandatory release intervals, and non-metallic self-retaining abdominal wall retraction systems achieved a substantial reduction in the incidence of nerve injury from 6.5% to 0.9%. These findings highlight the critical importance of meticulous surgical techniques in preventing iatrogenic nerve injuries. The systematic approach used in this study, focusing on simple yet effective interventions, resulted in a measurable reduction in this significant postoperative complication, thereby potentially improving patient outcomes.
